# Biomarkers of Dissolved Oxygen Stress in Oysters: A Tool for Restoration and Management Efforts

**DOI:** 10.1371/journal.pone.0104440

**Published:** 2014-08-12

**Authors:** Heather K. Patterson, Anne Boettcher, Ruth H. Carmichael

**Affiliations:** 1 Department of Marine Sciences, University of South Alabama, Mobile, Alabama, United States of America; 2 Dauphin Island Sea Lab, Dauphin Island, Alabama, United States of America; 3 Department of Biology, University of South Alabama, Mobile, Alabama, United States of America; Auckland University of Technology, New Zealand

## Abstract

The frequency and intensity of anoxic and hypoxic events are increasing worldwide, creating stress on the organisms that inhabit affected waters. To understand the effects of low dissolved oxygen stress on oysters, hatchery-reared oysters were placed in cages and deployed along with continuously recording environmental data sondes at a reef site in Mobile Bay, AL that typically experiences low oxygen conditions. To detect and measure sublethal stress, we measured growth and survival of oysters as well as expression of three biomarkers, heat shock protein 70 (HSP70), hypoxia inducible factor (HIF) and phospho-p38 MAP kinase, in tissues from juvenile and adult oysters. Survival rates were high for both juvenile and adult oysters. Expression levels of each of the 3 isoforms of HSP 70 were negatively correlated to dissolved oxygen (DO) concentrations, suggesting that HSP 70 is useful to quantify sublethal effects of DO stress. Results for HIF and phospho-p38 MAP kinase were inconclusive. Test deployments of oysters to assess expression of HSP 70 relative to environmental conditions will be useful, in addition to measuring abiotic factors, to identify appropriate sites for restoration, particularly to capture negative effects of habitat quality on biota before lethal impacts are incurred.

## Introduction

Restoration and management efforts are ongoing worldwide to enhance the area and function of oyster reefs to compensate for those that have gone functionally extinct [1]. Declines are linked to overharvesting, presence of non-native species, and disease as well as to degradation of environmental conditions, including increasing incidence of hypoxia [1], [2]. Pollack et al. [3] created a suitability index for oyster restoration based on salinity, temperature, turbidity, dissolved oxygen (DO) and depth, which demonstrated that identifying suitable sites for restoration is critical to success. This suitability index and associated reef quality index, however, could be taken a step further to include measurements of physiological stress, particularly to capture sublethal stress, further avoid species loss, and improve restoration success.

Anoxic and hypoxic conditions are pervasive and likely to be enduring because they are engendered by natural, as well as anthropogenic causes [4]. Low DO concentration conditions occur naturally due to water column stratification, normal biological processes and lack of wind mixing [5]. Anthropogenic low DO concentration conditions are typically related to increased inputs of land-derived nitrogen (N) to coastal waters that lead to phytoplankton blooms and their subsequent decomposition that consumes oxygen [6], [7]. Excessive or sustained low DO conditions contribute to loss of commercially important fishery stocks [8]–[10] and hinder associated restoration efforts [11], [12]. There are ongoing efforts world-wide to understand and manage the causes and effects of hypoxia on coastal ecosystems [7], [13]. Currently, the threshold for hypoxia is typically set at 2 mg L^−1^ DO concentration due to the significant impacts to ecosystems in terms of species diversity and abundance at and below this level, with 4 mg L^−1^ DO concentration set as the minimum level for maintenance of a healthy system (for review see [14]).

Low DO stress can have lethal and sublethal effects on many species, including oysters [15], [16]. Sublethal affects can lead to reduced growth, reduced feeding, and increased susceptibility to disease [16], [17]. Sublethal stress from low DO in relatively tolerant species such as oysters is not well understood, especially cumulative stress; given low DO concentration events are often episodic in nature [5], [8]. Several molecular biomarkers have been identified that may prove useful in examining low DO induced sublethal stress [18]. The term “biomarker” was first used in relation to environmental contamination, a biological response to chemical(s) given some amount of exposure [19]. Heat shock protein 70 (Hsp70) has been used broadly as a biomarker for environmental stress, and while first discovered in response to heat shock, it has been shown to respond to a variety of stressors [20]. Additional biomarkers for DO stress include hypoxia inducible factor (HIF) and phospho-p38 MAPK. Hsp70 has been increasingly used to study effects of a variety of abiotic stressors, but is not well studied in invertebrates [21].

Hsp70 is considered a molecular chaperone, it is responsible for maintaining folding, minimizing proteins aggregating in a non-native state and targeting denatured proteins for removal during normal cell functioning and during stress (reviewed in [22]–[25]). Hsp70 is highly conserved through evolutionary history [26]. In oysters, there are 3 isoforms of Hsp70 (Hsc77, Hsc72, Hsp69). The first two are constitutively expressed but there can be increased expression in response to stress, while the third is induced as a result of stress [27]. HIF is a transcription factor, which under low oxygen conditions leads to an increase in the transcription of genes involved in responses to low oxygen conditions [28], [29]. The signaling pathway involving HIF has been well studied in vertebrates, but initial studies in invertebrate systems suggested it might not be important or even present. The genetic sequence for HIF and associated transcriptional changes in response to hypoxia have been documented in the oyster *Crassostrea virginica* (Gmelin, 1791), suggesting that it may be a valuable marker for low DO concentration stress in oysters as in vertebrates [30]. p38 MAPKs are part of a group of protein kinases that are intermediaries between extracellular stress and the resulting increase in expression of other pathways in the cell nucleus [31], [32]. In marine mussels and snails, low DO concentration exposure was shown to increase p38 MAPK expression [33], [34]. In vertebrate systems the four isoforms of phospho-p38 MAPK responded to different stressors and not every isoform is present in every tissue type [31], with alpha and gamma isoforms implicated in hypoxia response [35]. The role of different isoforms in invertebrates has not been examined. To date there have been mixed results reported for the usefulness of biomarkers for hypoxia in invertebrates [36], [37], indicating that more detailed study is needed and suggesting that multiple biomarkers might be important.

**Figure 1:**
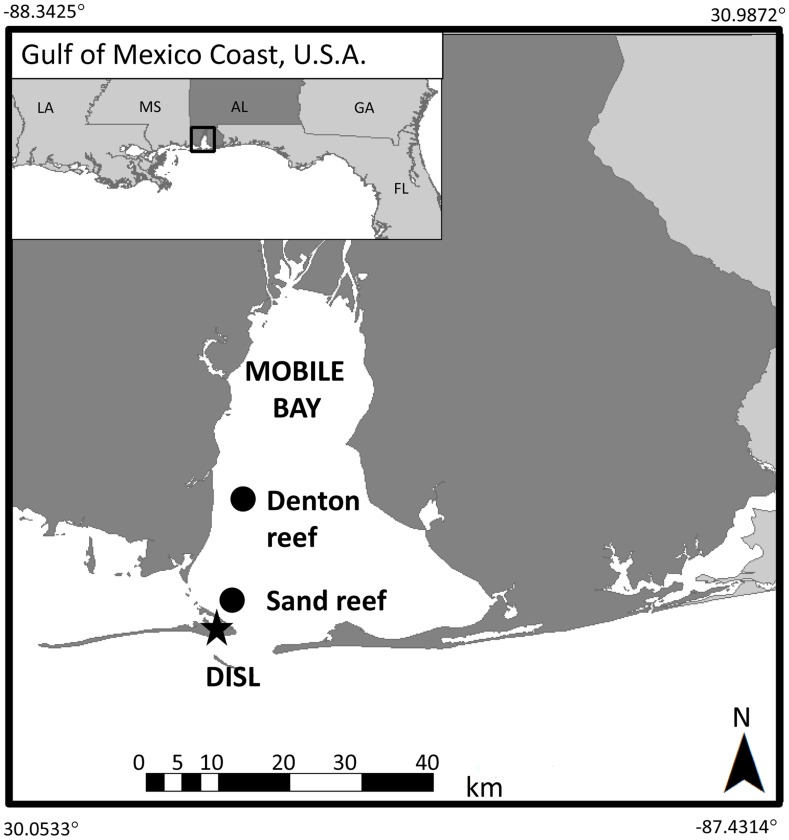
Sand Reef and Denton reef study site in Mobile Bay, AL near the Dauphin Island Sea Lab (DISL).

To examine the response of oysters to low DO concentration, a field study was performed in Mobile Bay, AL (Fig. 1), an area that experiences periodic extended low DO concentrations. To capture responses to low DO relative to ontogeny and relative to the range of bottom relief typical to reef sites, adult and juvenile oysters were deployed at two depths (0.1 m and 1.0 m from the sediment surface). Oysters were sampled at various time points, and traditional measurements of growth and survival were coupled to examination of the expression levels of 3 biomarkers, HSP70 (3 isoforms), HIF and p38 MAPK.

Mobile Bay presents an ideal location to study the effects of sublethal DO concentration stress on oysters. Although it experiences periodic low DO concentrations, there is still a standing stock of oysters and restoration efforts are currently underway [1], [38]. Historically oyster restoration in Mobile Bay has been dominated by shell planting with varied success [39]. More recently projects have used various types of cement blocks and focused on the economic benefits of oyster reef restoration to the Gulf of Mexico [40]. Native stocks and restoration projects will benefit from better understanding of the environmental conditions that lead to sublethal and lethal stress on oysters.

## Methods

### Oyster transplants

To assure that previous exposure was similar and that oysters were of the same genetic stock, oysters were obtained from the Auburn University Shellfish Hatchery and transplanted at Sand reef (Fig. 1), a historical oyster reef site in Mobile Bay, AL. Because the relief at this reef (largely remnant) varied gradually up to a height of 1.0 m above the sediment bottom across several meters, we simulated this natural relief by deploying caged oysters at fixed heights (0.1 m (bottom) and 1.0 m (top)) above the bottom on two existing pilings at each reef. This range of relief is also typical to oyster restoration projects [9]. To capture ontogenic variation in response to low DO conditions, 50 juvenile (40.1±0.3 mm) and 45 adult (62.8±0.4 mm) hatchery-reared oysters were placed in three replicate aquaculture cages (30 cm wide×40 cm long×10 cm deep). Cages were cleaned and oyster survival was noted every two weeks, at which time, up to 15 individuals of each age group were collected for growth measurement, mortality assessment, dry weight and protein analysis. To test cumulative effects, oysters were deployed for 87 days, thereby allowing the oysters to be exposed to several periods of low DO concentrations. To test for the co-occurring stressors of low DO concentration and low salinity, oysters were also deployed at nearby Denton reef, in Mobile Bay, AL (Fig. 1). Collection permits were attained from the Alabama Department of Conservation and Natural Resources.

### Environmental parameters

YSI 6600 data sondes were deployed to take measurements every 15 minutes, creating a 5 minute average of temperature, salinity, and DO. Sondes were deployed at the two cage depths, 1.0 m (top) and 0.1 m (bottom) off the bottom. Sondes were cleaned and calibrated ∼biweekly, using the manufacturer's standard methods for YSI 6-Series Sondes, one point saturated air method.

### Protein analyses

Gill, mantle and adductor muscle tissues were dissected from sampled oysters, frozen in liquid nitrogen, and stored at −80°C until analysis. Mantle and gill tissues have been shown to exhibit rapid response to stress [26]. We also chose to include adductor muscle because it is commonly sampled among all types of biological studies and it is important in the catch response, allowing the valves to open or close [41]. Tissues were extracted in lithium dodecyl sulfate (LiDS) sample buffer (1∶4 w∶v) (125 mM Tris pH 6.8, 10% glycerol, 2% LiDS); samples were centrifuged at 14,000×g for 10 minutes after which the supernatant was collected and re-centrifuged for another 10 minutes. Total protein concentration was determined using the BioRad DC assay (BioRad, Hercules, CA, USA) with bovine serum albumin standards. Proteins were analyzed by SDS-polyacrylamide gel electrophoresis. Samples of 100 µg total protein concentration were run on 8% gels at 100 V for 2.5 hours. Proteins were transferred to a nitrocellulose membrane (Amersham Hybond-ECL, GE Healthcare) at 100 V for 1 hour. Membranes were blocked for 1 hour with 50/50 Odyssey Buffer (LiCOR) and PBS (pH 7.4). Membranes were analyzed by immunoblotting with anti- HSP 70 antibodies (1∶10,000, Sigma St Louis, MO), HIF (created by Thermo Fisher from Genbank sequence HM441076, 1∶500 Waltham, MA) and p38 MAP kinase (1∶1,000, Cell Signaling Beverly, MA). Actin (1∶10,000, Neo Markers Fremont, CA) was used as a loading control, and an internal standard (anoxic treated mantle – mantle from oysters exposed to 4 days of anoxia) was run on all gels to allow between gel comparisons. Membranes were probed with GAR (1∶4,000) and GAM (1∶20,000) fluorescent secondary antibodies (LiCOR). Blots were viewed under infrared fluorescence (LiCOR, Lincoln, NE) and images analyzed with a LICOR Odyssey Imager and associated software (LiCOR, Lincoln, NE). Bands were quantified by drawing a box around and adjusted to the loading control (actin) and an internal standard (anoxic treated mantle). Band intensity is expressed as relative intensity.

### Statistical analysis

Minitab (version 16) was used for all statistical analyses. A GLM with date as a covariate was run on the environmental data and the growth and survival data. For the protein expression data all intensity values were square root and arc sin transformed before analysis. Liner regression analysis was used for protein expression versus 2-week average DO concentrations [27]. An α value of 0.05 was used for all tests.

## Results

### Site parameters

**Figure 2:**
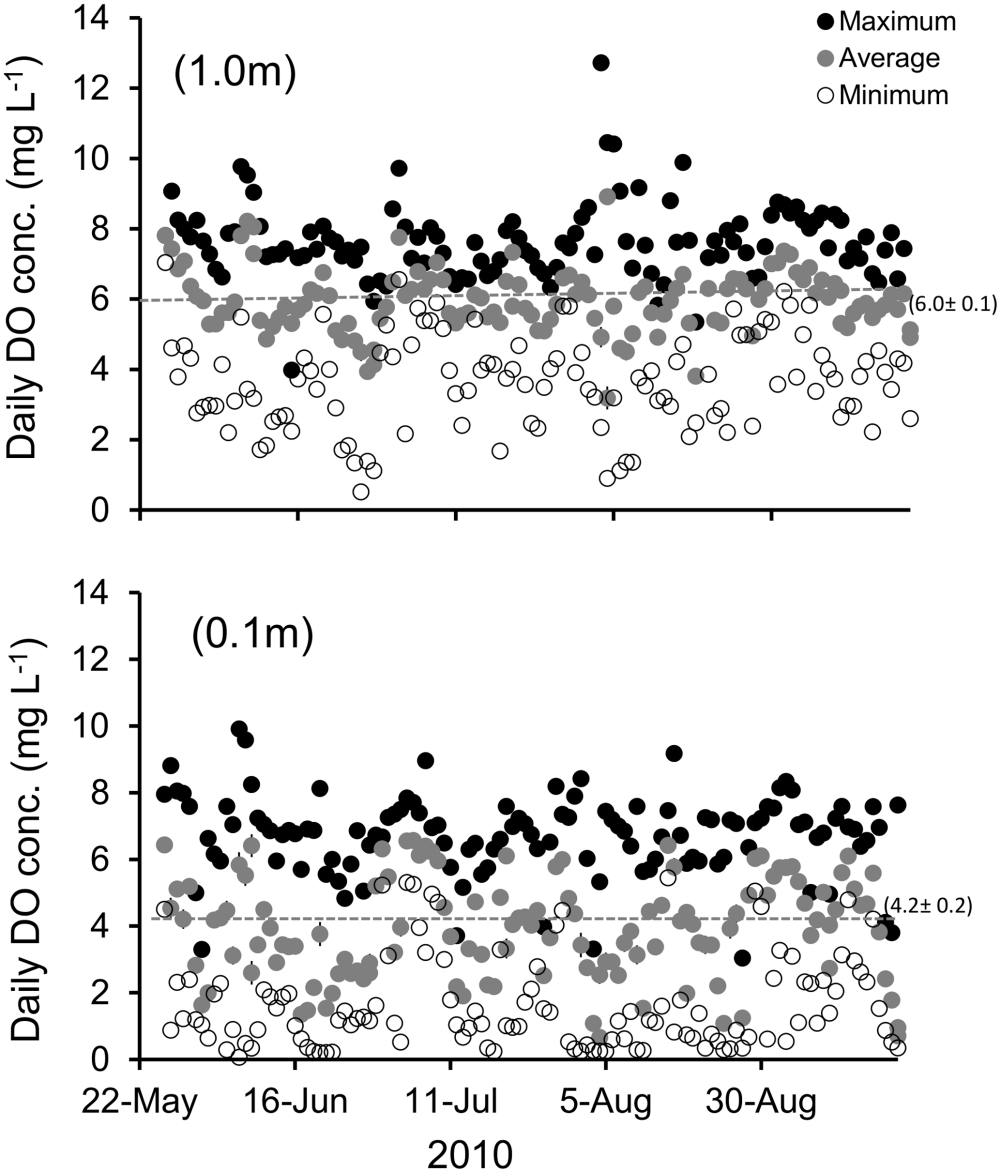
Daily maximum, average, and minimum dissolved oxygen (DO) concentrations for the top (1.0 m off bottom) and bottom (0.1 m off bottom) depth at Sand Reef, Mobile, AL.

**Figure 3:**
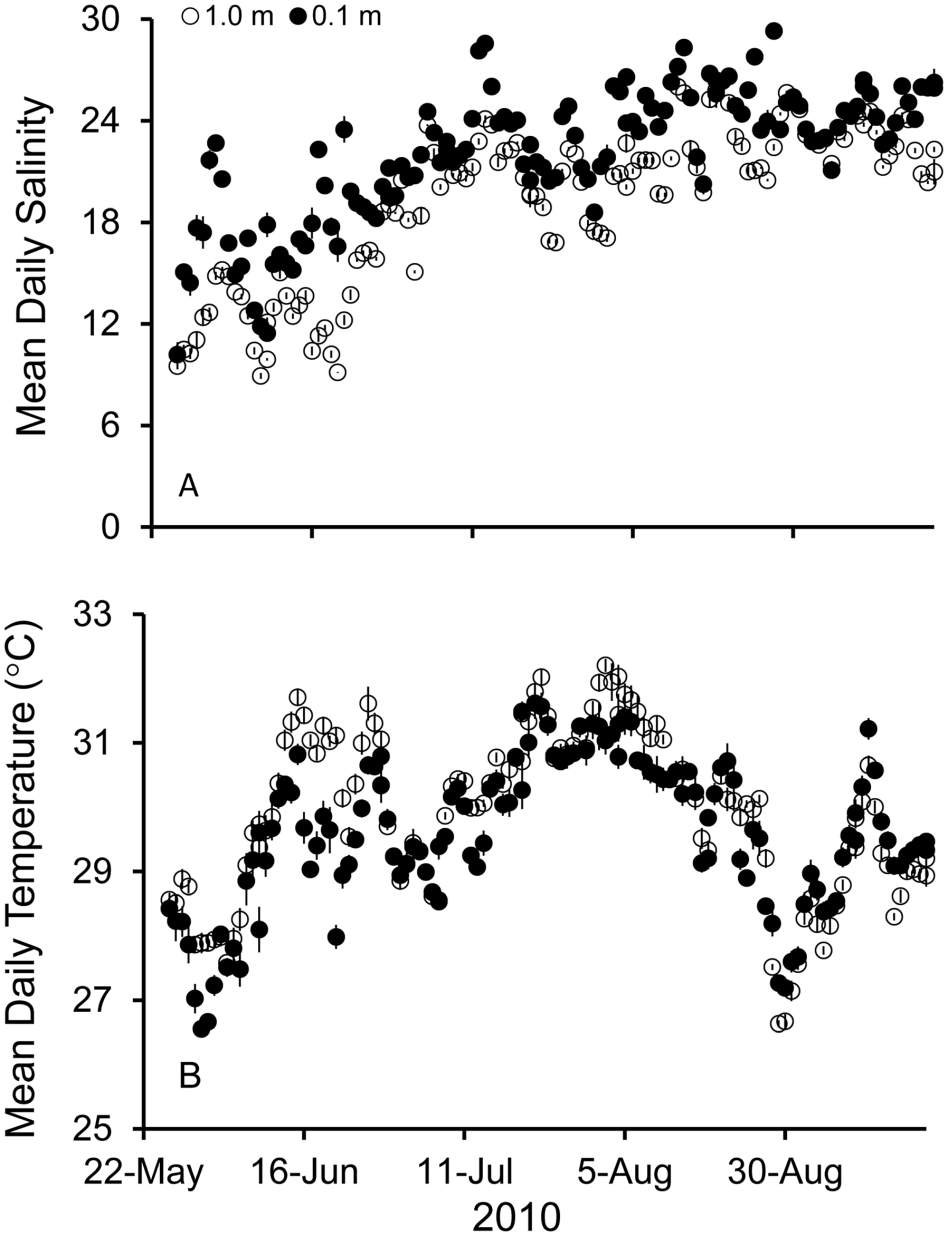
Daily mean salinity (A) and temperature (B) for the top depth (1.0 m off bottom) and the bottom depth (0.1 m off bottom) at Sand Reef, Mobile, AL.

Average daily DO concentrations differed through time at study sites on Sand reef in Mobile Bay, AL, with DO concentration at the top (1.0 m) significantly higher than at the bottom (0.1 m) depth (Fig. 2, p<0.001). Absolute DO concentrations ranged from 0.52 to 12.72 mg L^−1^ at the top and 0.07–9.91 mg L^−1^ at the bottom depth (Fig. 2). Average daily salinity also differed between the top (1.0 m) and bottom (0.1 m) depths (Fig. 3A, p<0.001), but temperature did not (Fig. 3B, p = 0.36). Salinity at the bottom depth ranged from 10.2 to 29.3 and at the top depth ranged from 8.9 to 26.7. Temperature ranged from 26.5–31.9°C overall. Sand reef had significantly higher salinity than comparison site Denton reef (GLM, *F* = 505.38, df = 1. p<0.001; Sand 20.68±0.02, Denton 14.76±0.02) and higher DO concentration (GLM, *F* = 24.67, df = 1, p<0.001 Sand 4.92±0.01 mg L^−1^, Denton 4.06±0.01 mg L^−1^).

### Growth and Survival

Juveniles had higher growth rates than adult oysters at their respective depths (Table 1). Oysters at the top depth had higher growth rates than those at the bottom depth (GLM, *F* = 27.63, df = 3. p<0.001). Survival did not differ with depth or between age classes and was greater than 90% at all depths and ages.

**Table 1:** Daily mean (± standard error) growth rates for juvenile and adult oysters and top and bottom depths, along with regression statistics.

	Growth rate (mm day^−1^)			
	Top (1.0 m)		Bottom (0.1 m)	
Juvenile	0.14±0.02	*F*_reg 1, 0.003_ = 40.82	0.12±0.02	*F*_reg 1, 0.003_ = 41.45
Adult	0.12±0.02	*F*_reg 1, 0.002_ = 35.80	0.07±0.03	*F*_reg 1, 0.034_ = 8.44

### Biomarkers

#### Hsp70

**Figure 4:**
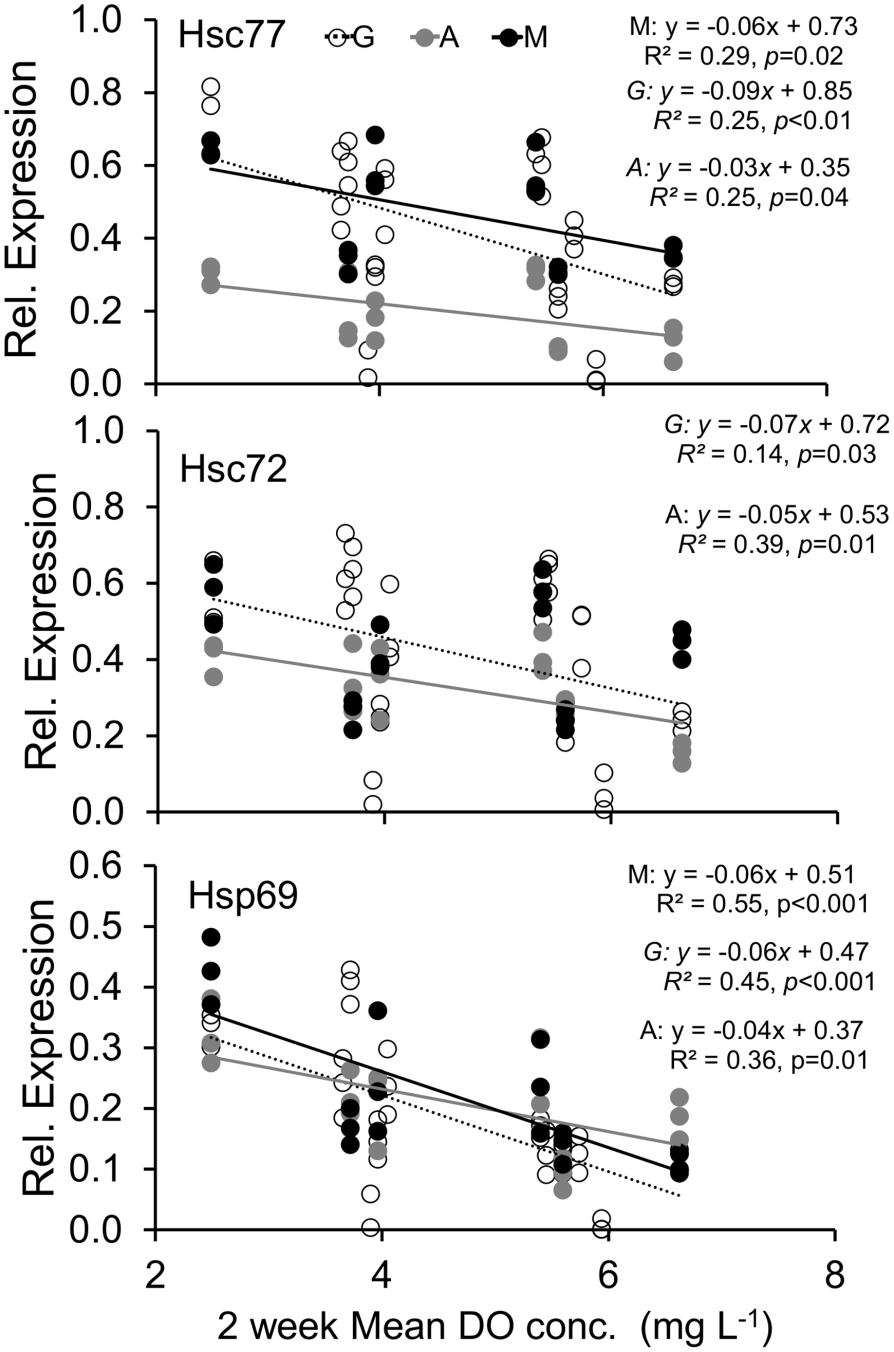
Regression analysis of juvenile gill (G, open circle, dashed line), adductor muscle (A, grey circle, grey line), and mantle (M, black circle, black line) tissue relative protein expression for the 3 isoforms of Hsp70 versus the average of previous 2 weeks daily dissolved oxygen (DO) concentrations. Regression lines are only present for significant regressions.

The three isoforms of Hsp70 were present in all tissues sampled from both age groups and transplant depths (example blot, Figure S1). For juveniles, 45% of the variation in relative protein expression of the inducible form of Hsp70 (Hsp69) in gill tissue was accounted for by DO concentration (Fig. 4, bottom), with higher expression corresponding to lower DO concentration. In the constitutive forms, there was also significantly higher expression with lower DO, although less of the variation was explained (25% and 14% respectively for Hsc 77 and 72, Fig. 4). Similar patterns were seen for expression of all three isoforms in juvenile adductor muscle (25, 39 and 36% for Hsc77, 72 and Hsp69 respectively, Fig. 4). However, with mantle tissue only Hsc77 and Hsp69 showed a relationship with DO concentration, with 29% (Hsc77) and 55% (Hsp 69) of variation in expression explained by DO concentration (Fig. 4).

**Figure 5:**
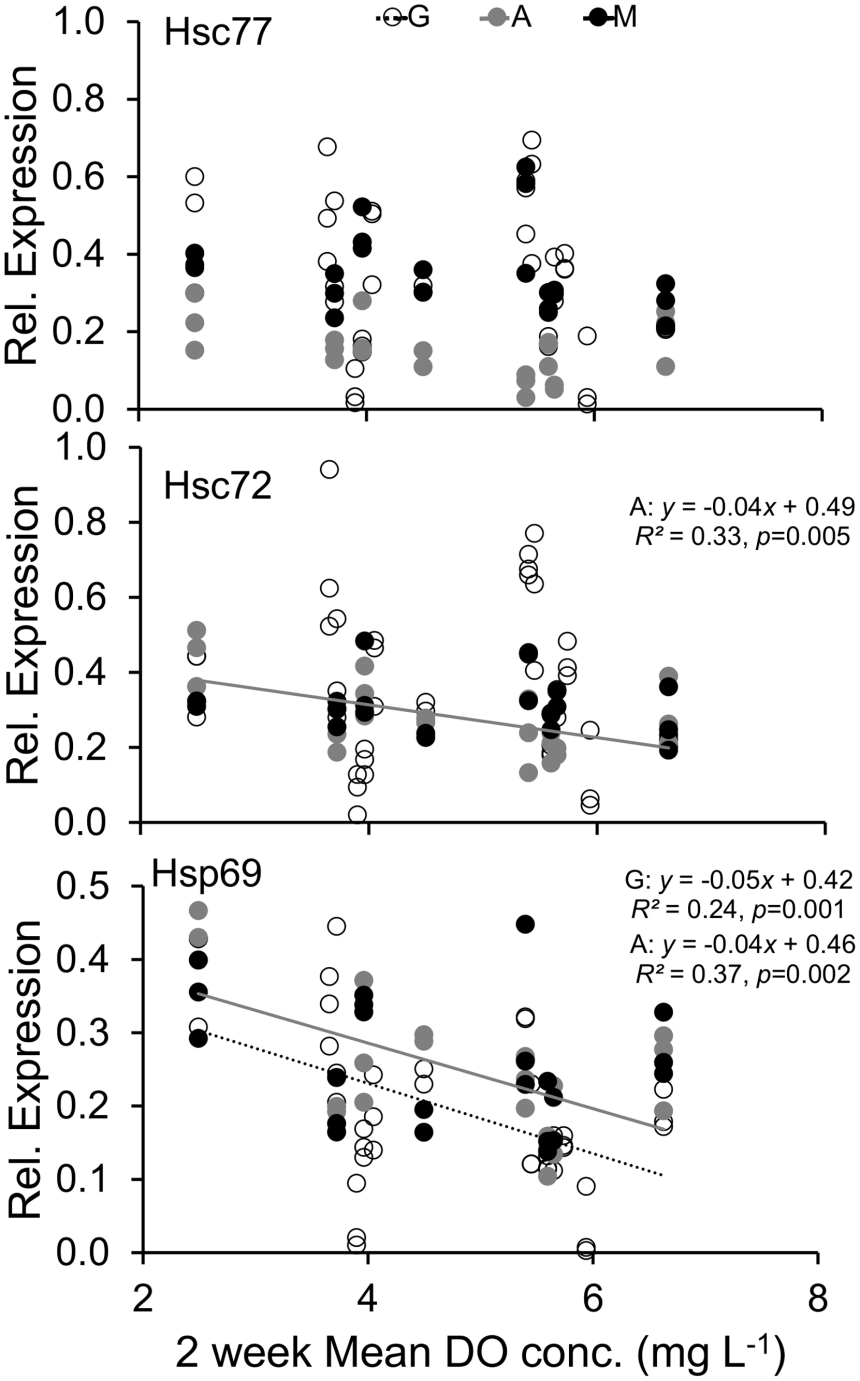
Regression analysis of adult gill (G, open circle, dashed line), adductor muscle (A, grey circle, grey line), and mantle (M, black circle, black line) tissue relative protein expression for the 3 isoforms of Hsp70 versus the average of previous 2 weeks daily dissolved oxygen (DO) concentrations. Regression lines are only present for significant regressions.

For adult oysters, 24% of Hsp69 expression in gill tissue was explained by DO concentration (Fig. 5, bottom). In adult gill tissue, there was no significant relationship between expression of the constitutive isoforms (Hsc77, Hsc72) and DO concentration (Fig. 5, top panels). In adult adductor muscle Hsc72 and Hsp69 expression showed a positive relationship with DO concentration (Fig. 5, middle and bottom). For the adult mantle tissue, there was no relationship between the expression of any of the Hsp70 isoforms and DO concentration (Fig. 5).

**Figure 6:**
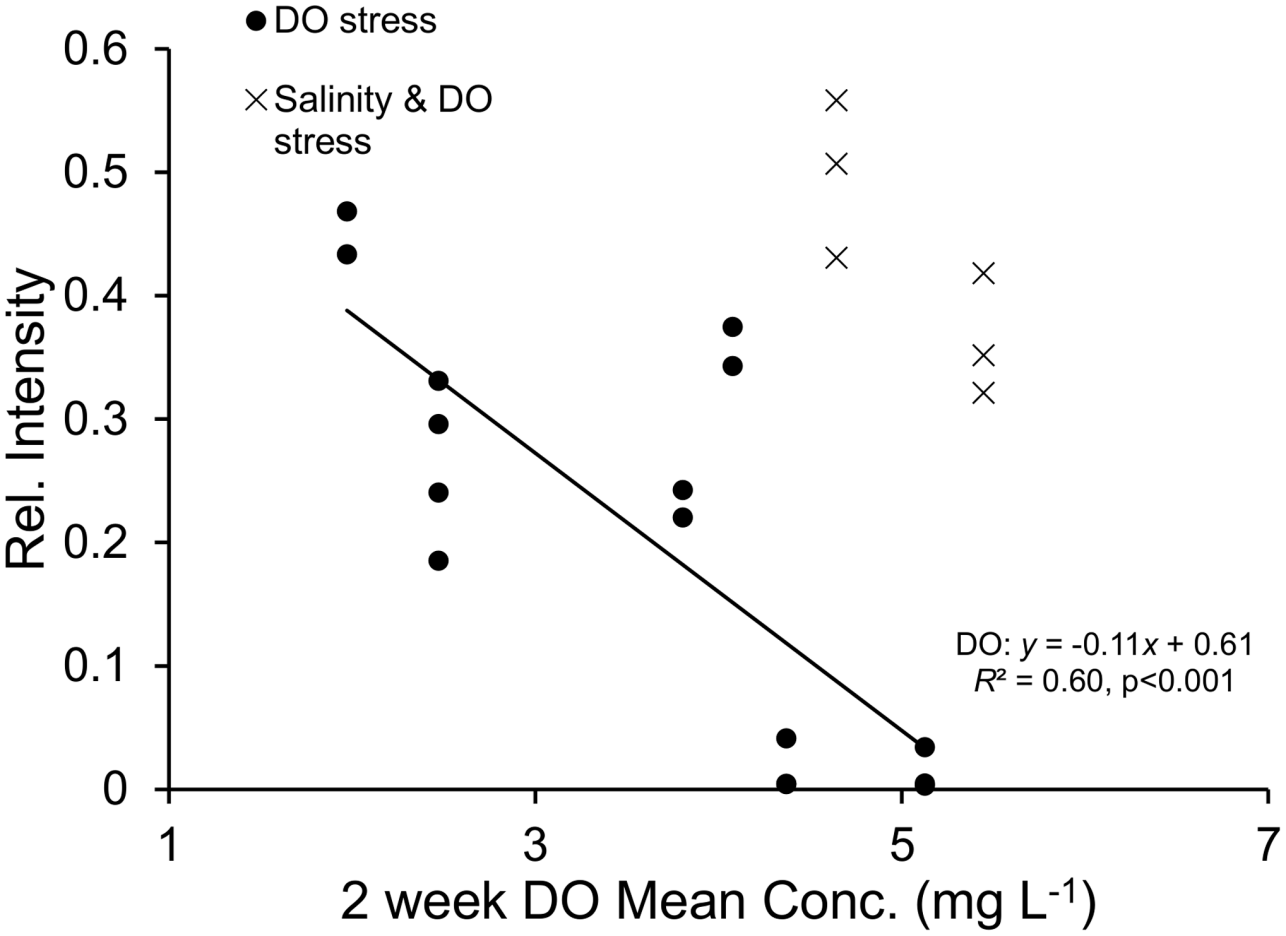
Regression analysis of juvenile gill tissue for the inducible Hsp70 isoform, Hsp69 versus the average of the previous 2 weeks daily dissolved oxygen (DO) concentrations. DO data from Denton reef (circles), and data when there was both salinity and DO stress as defined by [41] (X).

Low DO also accounted for 60% of the variation Hsp69 in juvenile gill tissue at nearby Denton reef. Interestingly, when oysters were subjected to dual stressors (low salinity and low DO at Denton reef), Hsp69 expression remains high even with higher DO concentration (Fig. 6).

#### HIF

We detected HIF expression, but did not observe any change in HIF expression in relation to different DO concentrations, regardless of age or depth (data not shown, Figure S2).

#### Phospho-p38 MAPK

We were not able to separate the alpha and beta isoforms, the combined isoform alpha/beta was expressed, along with the gamma isoform, in oysters from both depths, ages and in all three tissue types. We detected differences in expression between ages, depths and isoforms, but we found no pattern related to DO concentration or any other stressor (data not shown, Figure S3).

## Discussion

With the exception of periodic low DO concentrations (down to 3.2% saturation) most environmental parameters at study sites in Mobile Bay, AL were within the ranges suitable for oyster survival and growth [41], [42]. Accordingly oysters at our study sites survived well and had growth rates typical to the location and season [43], but showed physiological responses (protein expression) consistent with periodic stress associated with low DO concentrations. We found the best relationship between Hsp70 expression and the 2-week average DO concentration, indicating that the recent history of exposure to low DO through time is important to detect stress responses in oysters. Helmuth & Hofmann [44] found a similar pattern for temperature response. These data demonstrate the benefit of monitoring oysters in the field for several months while continuously measuring environmental attributes to test conditions oysters would experience in restoration programs.

There were differences in protein expression among the Hsp70 isoforms between the two age classes of oysters. There was greater expression of all three isoforms of Hsp70 in the juveniles in response to low DO concentration. However, for adults the primary effects were only seen for the inducible form Hsp69. Interestingly, there also were tissue-specific differences in protein expression between age classes. Such differences have been observed by other authors (e.g., [26]); however, the exact reason for these differences is not yet clear. Differences between age classes may indicate an ontogenetic effect due to either differences in relative growth rate or differences in physiological or cell functioning between age classes. Other authors have demonstrated that age can affect organism response to stress [45], [46]. These findings highlight the need to measure cumulative stress effects and consider endogenous factors (tissue type and age class) to identify a dominant environmental stressor.

Finding a biomarker that specifically responds to low DO conditions in oysters is a work in progress. We did not detect differences in expression of hypoxia inducible factor (HIF), a known hypoxia biomarker in other species, in relation to changes in DO concentration or with age in oysters, despite antibody detection of the protein in oysters and embryonic mouse lung (Figure S2). Our findings may result from detection of the constitutive expression and not expression of the different subunits as in vertebrate systems [47]–[49]. Kawabe & Yokoyoma [49] found changes in protein and m-RNA expression, using a different peptide that was not available when we performed this study. This peptide may be more appropriate for hypoxia investigations. We were able to detect phosphorylation of at least 3 isoforms of phospho-p38 MAPK, indicating the protein expression increased and that oysters were experiencing stress. We however, did not detect significant patterns in any of the isoforms, in either age class. Since DO concentration only accounted for up to 50% of the variation in Hsp70 isoform expression, the expression of the phosphorylated isoforms of p38-MAPK may be due to other stressors such as low salinity, infection, or environmental pollution [50], [51]. Many studies have detected a response to thermostress, contaminant exposure, and multiple stressors by induction of Hsp70 in oysters (e.g., [26], [27], [52]–[56]). Hsp70 responded to the dominant stressor in our system, DO concentration; based on our findings, Hsp70 isoforms, particularly Hsp69, show promise to indicate sublethal stress before traditional growth and survival measurements are affected.

Experiments to test site suitability prior to selection have been recognized as key to successful restoration of oyster reefs [3], [43], [57], [58]. The suitability index created by Pollack et al. [3] to aid in oyster restoration site management, stressed the importance of adaptive management strategies for site selection. In addition to the information suggested by Pollack et al. [3] (abiotic and oyster abundance), using stress proteins as broad response biomarkers has been recommended for initial environmental monitoring [59], [60]. We have shown that Hsp70 has the ability to respond to an abiotic stressor (low DO concentration) within two weeks, even in relatively stress-tolerant bivalves [61], prior to detectable effects on growth or survival. Understanding sublethal stress to predict and avoid species loss has broad applications, and has been identified as important to management of communities from recreational fisheries to coral reefs as well as to contaminant monitoring [62]–[64]. Our data support the idea that protein biomarkers, measured in sentinel species such as oysters, provide a promising approach to identify suitable sites for restoration, particularly to capture negative effects of habitat quality on biota before lethal impacts are incurred.

## Supporting Information

Figure S1 **Example blot of Hsp70 showing the 3 isoforms (Hsc77, Hsc72, Hsp69) and Actin in Sand Bottom (SB) Adult mantle and AM (adductor muscle) tissues and in the internal standard (Std).**
(TIF)

Figure S2 **Example blot of HIF with mouse embryonic lung (lung), juvenile gill tissue from hypoxic site 6/9/10 (DBJ), juvenile gill tissue from normoxic site 8/4/10 (STJ) and internal standard (STD).**
(TIF)

Figure S3 **Example blot of phopho-p38 MAPK showing the gamma (γ) and alpha/beta (α/β) isoforms in adult and juvenile gill tissue 9/21/10 for the top and bottom depths.**
(TIF)
