# Supplementary figures and images for: Biomarkers of Dissolved Oxygen Stress in Oysters: A Tool for Restoration and Management Efforts
Source: PLoS One. 2014 Aug 12;9(8):e104440. doi: 10.1371/journal.pone.0104440 (PMC4130543; doi:10.1371/journal.pone.0104440)

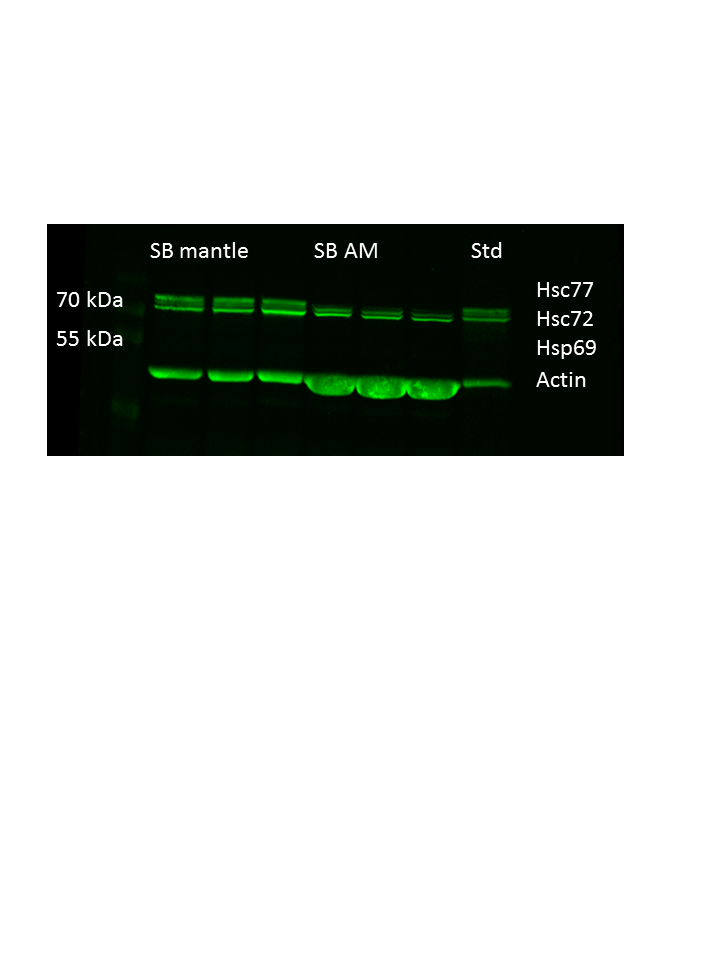

Supplement: Figure S1 — Example blot of Hsp70 showing the 3 isoforms (Hsc77, Hsc72, Hsp69) and Actin in Sand Bottom (SB) Adult mantle and AM (adductor muscle) tissues and in the internal standard (Std). (TIF) [file pone.0104440.s001.tif]

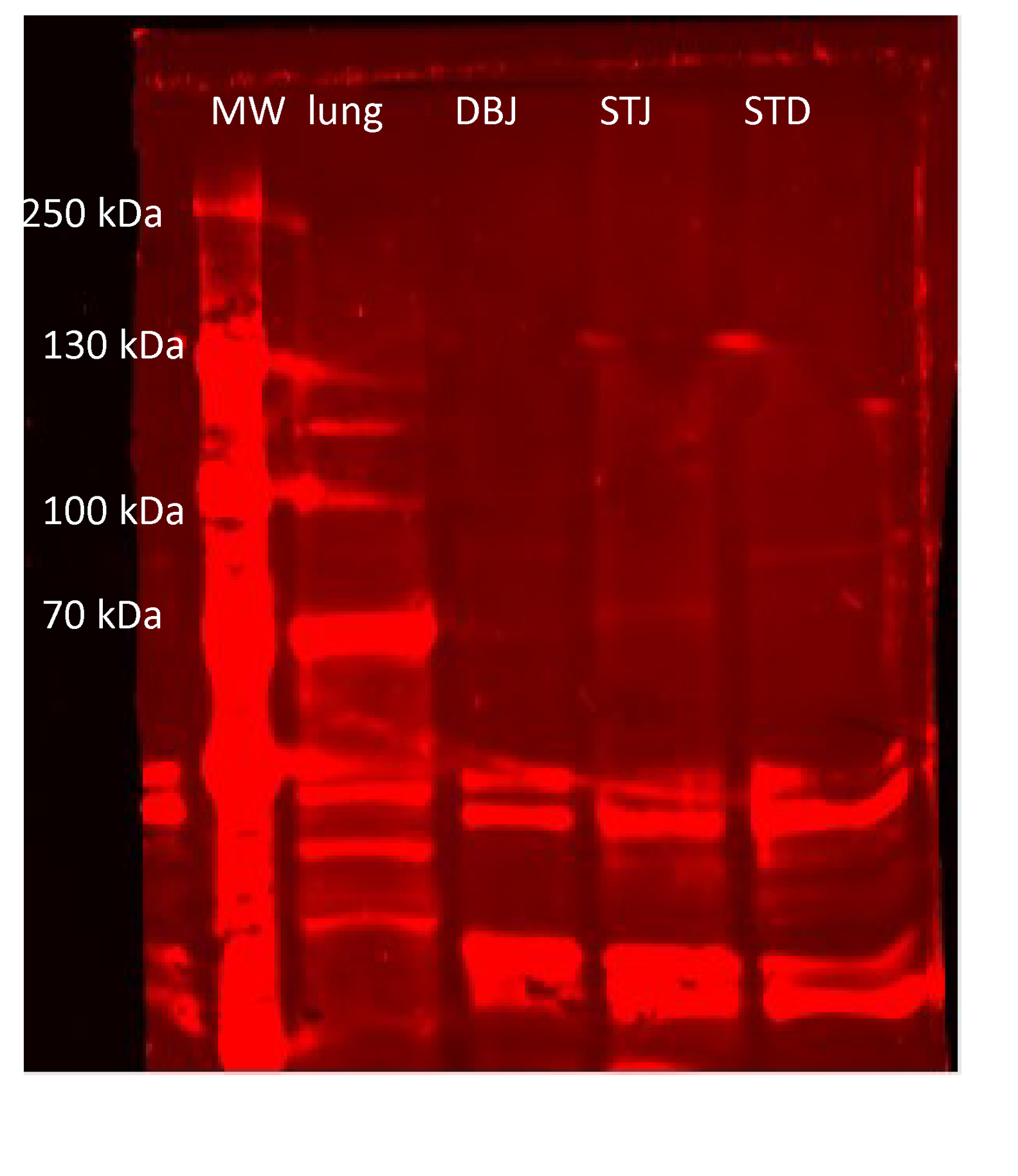

Supplement: Figure S2 — Example blot of HIF with mouse embryonic lung (lung), juvenile gill tissue from hypoxic site 6/9/10 (DBJ), juvenile gill tissue from normoxic site 8/4/10 (STJ) and internal standard (STD). (TIF) [file pone.0104440.s002.tif]

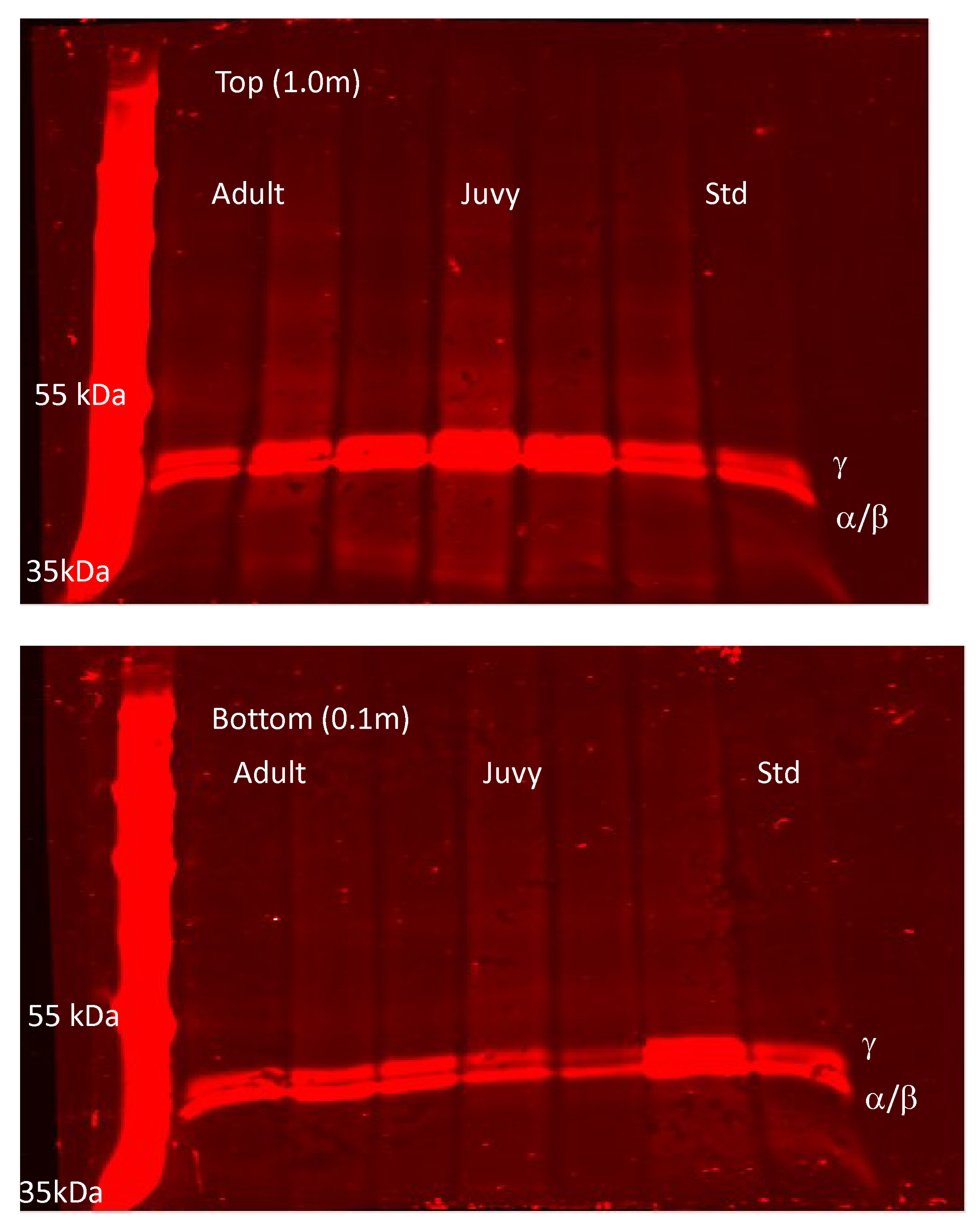

Supplement: Figure S3 — Example blot of phopho-p38 MAPK showing the gamma (γ) and alpha/beta (α/β) isoforms in adult and juvenile gill tissue 9/21/10 for the top and bottom depths. (TIF) [file pone.0104440.s003.tif]
